# Azithromycin Resistance in Shigella spp. in Southeast Asia

**DOI:** 10.1128/AAC.01748-17

**Published:** 2018-03-27

**Authors:** Thomas C. Darton, Ha Thanh Tuyen, Hao Chung The, Paul N. Newton, David A. B. Dance, Rattanaphone Phetsouvanh, Viengmon Davong, James I. Campbell, Nguyen Van Minh Hoang, Guy E. Thwaites, Christopher M. Parry, Duy Pham Thanh, Stephen Baker

**Affiliations:** a The Hospital for Tropical Diseases, Wellcome Trust Major Overseas Programme, Oxford University Clinical Research Unit, Ho Chi Minh City, Vietnam; b Department of Infection, Immunity and Cardiovascular Disease, University of Sheffield Medical School, Sheffield, United Kingdom; c Lao-Oxford-Mahosot Hospital-Wellcome Trust Research Unit, Vientiane, Laos; d Centre for Tropical Medicine, Nuffield Department of Clinical Medicine, Oxford University, Oxford, United Kingdom; e Faculty of Infectious and Tropical Diseases, London School of Hygiene and Tropical Medicine, London, United Kingdom; f Clinical Sciences, Liverpool School of Tropical Medicine, Liverpool, United Kingdom; g School of Tropical Medicine and Global Health, Nagasaki University, Nagasaki, Japan; h Department of Medicine, University of Cambridge, Cambridge, United Kingdom

**Keywords:** Shigella, Southeast Asia, azithromycin, breakpoints, genome analysis, resistance

## Abstract

Infection by Shigella spp. is a common cause of dysentery in Southeast Asia. Antimicrobials are thought to be beneficial for treatment; however, antimicrobial resistance in Shigella spp. is becoming widespread. We aimed to assess the frequency and mechanisms associated with decreased susceptibility to azithromycin in Southeast Asian Shigella isolates and use these data to assess appropriate susceptibility breakpoints. Shigella isolates recovered in Vietnam and Laos were screened for susceptibility to azithromycin (15 μg) by disc diffusion and MIC. Phenotypic resistance was confirmed by PCR amplification of macrolide resistance loci. We compared the genetic relationships and plasmid contents of azithromycin-resistant Shigella sonnei isolates using whole-genome sequences. From 475 available Shigella spp. isolated in Vietnam and Laos between 1994 and 2012, 6/181 S. flexneri isolates (3.3%, MIC ≥ 16 g/liter) and 16/294 S. sonnei isolates (5.4%, MIC ≥ 32 g/liter) were phenotypically resistant to azithromycin. PCR amplification confirmed a resistance mechanism in 22/475 (4.6%) isolates (*mphA* in 19 isolates and *ermB* in 3 isolates). The susceptibility data demonstrated the acceptability of the S. flexneri (MIC ≥ 16 g/liter, zone diameter ≤ 15 mm) and S. sonnei (MIC ≥ 32 g/liter, zone diameter ≤ 11 mm) breakpoints with a <3% discrepancy. Phylogenetic analysis demonstrated that decreased susceptibility has arisen sporadically in Vietnamese S. sonnei isolates on at least seven occasions between 2000 and 2009 but failed to become established. While the proposed susceptibility breakpoints may allow better recognition of resistant isolates, additional studies are required to assess the impact on the clinical outcome. The potential emergence of azithromycin resistance highlights the need for alternative options for management of Shigella infections in countries where Shigella is endemic.

## INTRODUCTION

Organisms of the bacterial genus Shigella are a common cause of moderate to severe diarrhea and dysentery in children attending day care facilities, those living in resource-limited settings, and travelers to such areas (1–5). In many low- to middle-income countries (LMICs), such as Vietnam, endemic shigellosis is now predominantly caused by Shigella sonnei. Sustained antimicrobial pressure in LMICs has led to the emergence of resistance to the antimicrobials used for treating shigellosis (6, 7). In Southeast Asia, antimicrobial resistance (AMR) in the shigellae is largely being driven by the expansion of a specific S. sonnei lineage, which is known as global lineage III (8).

AMR within the genus Shigella is a problem for clinical management (9, 10). The treatment of Shigella infections with antimicrobials is recommended by most clinical guidelines, predominantly to reduce the risk of onward transmission and disease complications. WHO currently recommends ciprofloxacin as the first-line treatment, with pivmecillinam, ceftriaxone, and azithromycin being alternative options. However, Shigella spp. are adept at acquiring AMR genes and plasmids, and reports of multidrug-resistant (MDR) lineages or isolates with reduced susceptibility to fluoroquinolones and third-generation cephalosporins are increasing globally (11, 12).

Some recent recommendations have advocated the oral azalide antimicrobial azithromycin as an alternative treatment for shigellosis, particularly for infections caused by MDR organisms or when fluoroquinolones are inappropriate (9, 13). Clinical evidence for the efficacy of azithromycin in treating shigellosis is limited (14, 15), and there are presently no suitable clinically derived susceptibility breakpoints to facilitate the laboratory identification of Shigella spp. exhibiting azithromycin nonsusceptibility. Recently updated CLSI guidelines suggest epidemiological cutoff values (ECVs) of MICs of ≥16 mg/liter and ≥32 mg/liter to the categories non-wild-type S. flexneri and S. sonnei, respectively (16). Data supporting these guidelines are limited and principally originate from reports of an international outbreak of S. flexneri serotype 3a among men who have sex with men (MSM) (17–19). Here, we aimed to assess the frequency and mechanisms of Shigella species isolates with decreased susceptibility to azithromycin in Southeast Asia, a setting where fluoroquinolone and third-generation cephalosporin resistance has become common. Additionally, using a large data set from Vietnam and Laos spanning 18 years, we aimed to calculate suitable breakpoints for assessing Shigella susceptibility to azithromycin.

## RESULTS

### Decreased susceptibility to azithromycin in Shigella spp. in Southeast Asia.

Data from a total of 517 Shigella isolates (198 S. flexneri isolates, 308 S. sonnei isolates, and 11 isolates of other Shigella spp.) collected between 1994 and 2012 in Vietnam (6 studies, 472 isolates) and Laos (45 isolates) (Table 1) were available for antimicrobial susceptibility analysis. In this collection of organisms, 180/198 (91%) S. flexneri isolates were defined as being MDR (resistant to ≥3 classes of antimicrobials), 3/196 (2%) were resistant to ceftriaxone, and 78/196 (40%) were resistant to nalidixic acid. In contrast, significantly fewer S. sonnei isolates were MDR (181/308, 59%; *P* < 0.0001), while a greater proportion exhibited resistance to ceftriaxone (92/307, 30%; *P* < 0.0001) and nalidixic acid (174/307, 69%; *P* = 0.0003) (20).

**TABLE 1:** Origin of Shigella isolates and frequency of selected resistance azithromycin markers^*a*^

Country/study code^*a*^	Period of study	No. of isolates of the following Shigella species:				No. of isolates with the following antimicrobial resistance markers/total no. of isolates tested (%)^*b*^			
		S. flexneri	S. sonnei	Other	Total	DSA	NAL	CRO	MDR
Vietnam/MS	1994–1998	58	22	0	80	3/70 (4.3)	1/80 (1.3)	0/80 (0)	57/80 (72.5)
Vietnam/DE	2000–2002	42	62	8^*c*^	112	10/93 (10.8)	32/111 (28.8)	1/111 (0.9)	80/112 (71.4)
Laos	2006–2012	35	9	1^*d*^	45	0/45 (0)	14/45 (31.1)	0/45 (0)	34/45 (75.6)
Vietnam/EG	2007–2008	30	78	2^*e*^	110	4/104 (3.8)	75/108 (69.4)	22/108 (20.3)	96/110 (87.3)
Vietnam/Hué	2008–2010	21	37	0	58	1/56 (1.8)	27/58 (46.6)	7/58 (12.0)	24/58 (41.4)
Vietnam/AV	2009–2010	4	58	0	62	3/61 (4.9)	58/62 (93.5)	47/62 (75.8)	52/62 (83.9)
Vietnam/KH	2009–2010	8	42	0	50	1/50 (2.0)	47/50 (94.0)	18/50 (36.0)	25/50 (50)
Total		198	308	11	517	22/479 (4.8)	254/514 (49.4)	95/514 (18.5)	368/517 (71.2)

a The study code is described in reference 6.

b DSA, decreased sensitivity to azithromycin (S. flexneri MIC ≥ 16 mg/liter; S. sonnei MIC ≥ 32 mg/liter); NAL, nalidixic acid-resistant organism (zone diameter < 19 mm); CRO, ceftriaxone-resistant organism (zone diameter < 23 mm); MDR, multidrug resistant, which includes isolates intermediate or resistant to ≥3 classes of the following antimicrobials: penicillins (ampicillin), cephems (ceftriaxone), folate inhibitors (trimethoprim), phenicols (chloramphenicol), tetracyclines (tetracycline), quinolones (specifically, nalidixic acid resistance), aminoglycosides (gentamicin).

c One S. boydii isolate, one S. dysenteriae isolates, and six isolates for which the species was not available.

d S. boydii.

e Two S. boydii isolates.

**FIG 1:**
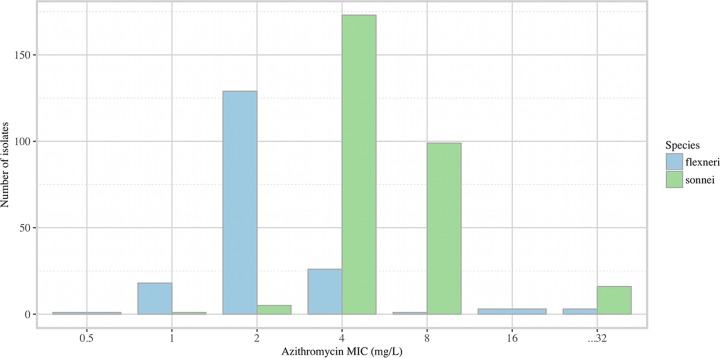
Distribution of azithromycin MICs for S. flexneri and S. sonnei in Southeast Asia. The histograms show the number of S. sonnei and S. flexneri isolates collected in 7 studies performed in Southeast Asia between 1994 and 2012 exhibiting different MICs against azithromycin.

From the 517 Shigella isolates collected over the defined period, 479 were recovered and available for azithromycin susceptibility testing; 181/479 (37.8%) were S. flexneri isolates, 294/479 (61.4%) were S. sonnei isolates, and 4/479 (0.8%) were isolates belonging to other Shigella species (which were not considered further). The distributions of the azithromycin MICs of the 475 Shigella isolates collected over the sampling period are shown in Fig. 1. The combined MIC_50_ for azithromycin was 4 mg/liter (MIC_90_, 8 mg/liter); the S. sonnei isolates exhibited a higher range of MIC values (interquartile range [IQR], 4 to 8 mg/liter) than the S. flexneri isolates (IQR, 2 to 4 mg/liter). The proportion of S. flexneri isolates with an MIC of ≥16 mg/liter was 6/181 (3.3%; 95% confidence interval [CI], 1.4 to 7.4%), whereas the proportion of S. sonnei isolates with an MIC of ≥32 mg/liter was 16/294 (5.4%; 95% CI, 3.2 to 8.9%) (*P* > 0.05).

### Genes conferring decreased susceptibility to azithromycin.

Isolates were screened by PCR amplification for the macrolide resistance genes *ermA*, *ermB*, *ermC*, *mphA*, *mphB*, *ereA*, *ereB*, *msrA*, and *mefA*, which encode antimicrobial efflux mechanisms. Nucleic acid extractions from 19/475 (4.0%) isolates (14 S. sonnei and 5 S. flexneri isolates) generated an amplicon for *mphA* (Table 2). The majority of these organisms had azithromycin MICs of ≥32 mg/liter with a corresponding zone of inhibition of ≤14 mm; three S. flexneri isolates had azithromycin MICs of 16 mg/liter and zone sizes of 11 and 12 mm (2 isolates) to a 15-μg azithromycin disc. A further three organisms produced *ermB* amplicons (3/475, 0.6%). The only *ermB* amplification-positive S. flexneri isolate had a lower MIC (16 mg/liter) and a larger inhibition zone size (12 mm) than the two S. sonnei isolates (MIC, 32 mg/liter; zone size, 9 mm). These data suggest that S. sonnei and S. flexneri exhibit different distributions of MICs when harboring the *mphA* and/or *ermB* gene.

**TABLE 2:** Source and microbiological and genotypic characteristics of Shigella isolates with decreased susceptibility to azithromycin^*a*^

Isolate identifier	Organism	Yr	Age (yr)	Azithromycin susceptibility		Resistance gene	ESBL^*b*^ producer
				MIC (mg/liter)	Zone diam (mm)		
MS025	S. flexneri 2a	1994–1998	0.75	32	11	*mphA*	−
MS052	S. flexneri	1994–1998	0.83	16	14	*mphA*	−
MS055	S. flexneri 6	1994–1998	0.92	512	6	*mphA*	−
DE0088	S. sonnei	2000	4.00	512	6	*mphA*	−
DE0105	S. sonnei	2000	1.50	512	6	*mphA*	−
DE0108	S. sonnei	2000	1.50	512	6	*mphA*	−
DE0185	S. sonnei	2000	0.67	512	6	*mphA*	−
DE0199	S. sonnei	2000	2.42	512	6	*mphA*	−
DE0490	S. sonnei	2000	1.67	512	6	*mphA*	−
DE0579	S. sonnei	2001	4.00	512	6	*mphA*	−
DE0885	S. sonnei	2001	3.00	512	6	*mphA*	−
DE0891	S. sonnei	2001	1.50	128	6	*mphA*	−
DE1336	S. sonnei	2002	1.92	512	6	*mphA*	−
EG0094	S. sonnei	2007	2.58	256	6	*mphA*	−
EG0352	S. sonnei	2007	2.50	256	6	*mphA*	−
EG0419	S. flexneri 2a	2007	1.92	16	12	*ermB*	−
EG0430	S. sonnei	2008	3.00	32	9	*ermB*	+
Hué 49	S. flexneri	2009	4.00	128	6	*mphA*	−
KH 39	S. flexneri	2009	0.75	16	12	*mphA*	−
20094	S. sonnei	2010	1.42	32	9	*ermB*	+
20343	S. sonnei	2010	1.58	512	6	*mphA*	+
30295	S. sonnei	2010	1.75	512	6	*mphA*	+

a All isolates were MDR.

b ESBL, extended spectrum β-lactamase.

### Determining disc susceptibility breakpoints for azithromycin.

CLSI recently provided ECVs for determining azithromycin resistance in S. flexneri (disc diffusion and MIC) and S. sonnei (MIC only) (16). While ECVs are not generally recommended for determining clinical susceptibility breakpoints, we used the same criteria in our data set, given that clinical data on azithromycin usage were not available. We aimed to determine whether the CLSI cutoff values could be used to determine suitable disc diffusion breakpoints for S. sonnei. Azithromycin disc inhibition zone sizes were available for 181 S. flexneri and 294 S. sonnei isolates. A regression analysis for determining the suitability of the use of MIC data to extrapolate disc diffusion breakpoints demonstrated a significant correlation between the MIC and the disc diffusion zone size for S. flexneri (rho, −0.845; *P* < 0.0001, Spearman's rank correlation coefficient) and, to a lesser extent, for S. sonnei (rho, −0.649; *P* < 0.001).

**FIG 2:**
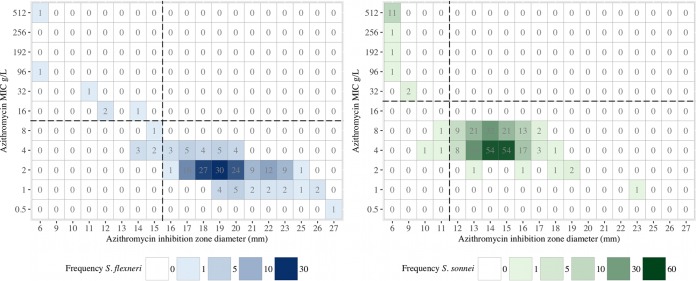
Relationship between azithromycin MIC and inhibition zone size in Southeast Asian Shigella spp. The plots show the relationship between inhibition zone size (*x* axis) and the MIC (*y* axis) for azithromycin in S. flexneri (left) and S. sonnei (right). The squares are colored with respect to the number of isolates in each group, and the number of isolate in each group is additionally provided.

For S. flexneri, a breakpoint zone size of ≤15 mm against a 15-μg azithromycin disc exhibited good discrimination to identify nonsusceptible isolates. Using an error rate-bounding method, a 3% major error rate was found, and with a ≤15-mm breakpoint, there were no very major or minor errors compared to the results obtained with an MIC of ≤8 mg/liter (Table 3; Fig. 2), thereby fulfilling CLSI recommendations (21). In contrast, while the ECV MIC threshold of ≥32 mg/liter appeared to define nonsusceptible S. sonnei isolates, no clear demarcation in disc diffusion zone size measurements was observed (Fig. 2). The largest azithromycin zone of inhibition in the S. sonnei isolates with a known azithromycin resistance mechanism was 9 mm. We aimed to identify the largest zone size concordant with a permissible CLSI error rate. We determined that a cutoff of ≤11 mm resulted in an acceptable discrepancy rate (Table 3), whereas one of ≤12 mm resulted in a 6.5% major error rate.

**TABLE 3:** Discrepancy rates of false-susceptible and false-resistant isolates detected using proposed breakpoint criteria and an error rate-bounding method

Organism (breakpoint [g/liter])	MIC range^*a*^	No. of isolates	No. (%) of discrepancies	
			Very major	Major
S. flexneri (≤8)	≥R + 1	3	0	NA^*b*^
	R + S	4	0	1 (25)
	≤S + 1	191	NA	5 (2.6)
	Total	198	0	6 (3.0)
S. sonnei (≤16)	≥R + 1	14	0	NA
	R + S	2	0	0
	≤S + 1	292	NA	3 (1.0)
	Total	308	0	3 (1.0)

a R, nonsusceptible MIC; S, susceptible MIC; +1, +1 MIC dilution (according to CLSI guidelines).

b NA, not applicable.

### Plasmid structures and phylogenetic context of azithromycin-resistant Shigella sonnei.

**FIG 3:**
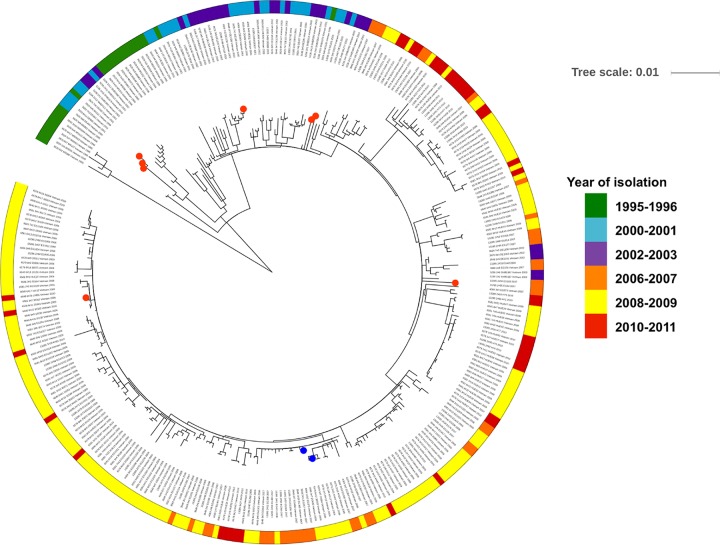
Phylogenetic tree of 261 S. sonnei genomes (global lineage III) and an additional 54 genomes of isolates collected during the same period (1995 to 2011) in Southeast Asia. The tree was constructed through the use of 2,812 chromosomal SNPs. Phylogenetic reconstruction was performed using multiple-sequence alignments of the SNPs by maximum likelihood-based phylogenetic inference; the tree was displayed and annotated using the iTOL tool. The year/period of isolation is highlighted in the outer ring, and the organisms with reduced susceptibility to azithromycin are identified; *mphA*-positive isolates are highlighted in red, and *ermB*-positive isolates are highlighted in blue.

As observed previously, phylogenetic analyses confirmed that all Vietnamese S. sonnei isolates whose genomes have been sequenced belong to the same clade as global lineage III (22). Investigation of the accessory genome confirmed that resistance to azithromycin was mediated by either *ermB* or *mphA* in 16 of these sequenced S. sonnei isolates (Fig. 3). Two of the 16 azithromycin-resistant isolates carried an *ermB* gene; the remaining 14 carried an *mphA* gene. Notably, unlike the phenotypes of reduced susceptibility to fluoroquinolones and resistance to third-generation cephalosporins (23), these azithromycin resistance genes were not restricted to individual sublineages or clonal expansions. Indeed, we estimated that between 2001 and 2008 *ermB* was acquired independently on at least two separate occasions, while *mphA* was acquired on at least five separate occasions, forming a small subclade of azithromycin-resistant organisms in two instances (Fig. 3). However, these azithromycin resistance genes were transient and appeared not to be maintained within the population.

**FIG 4:**
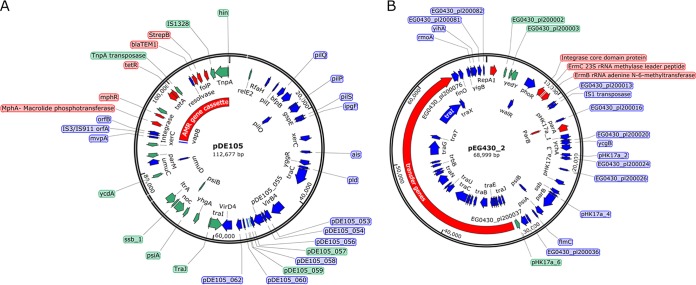
Maps of azithromycin resistance plasmids pDE105 (A) and pEG430_2 (B) from Vietnamese S. sonnei isolates. The coding sequences are numbered consecutively, and notable genes/regions, including DNA transfer, replication, and antimicrobial resistance regions and the azithromycin resistance genes (*ermB* and *mphA*), are highlighted. The size of each plasmid is shown in the center.

Additional *in silico* analysis of the azithromycin resistance plasmids demonstrated that *ermB* is associated with two different plasmid structures; S. sonnei 20094 harbored an IncFI plasmid (p20094), and S. sonnei EG430 carried an IncFII plasmid (pEG430-2). The IncFI plasmid (p20094) was assembled and found to be approximately 82 kb in size, sharing 99% DNA sequence identity with pEG356 (GenBank accession no. FN594520.1), which we previously characterized in Vietnamese S. sonnei isolate EG356 (23). Similar to plasmid pEG356, p20094 carried a *bla*_CTX-M-24_ gene downstream of an IS*Ecp1* element. However, this replicon additionally contained an IS*CR3* insertion sequence encompassing both the *ermB* and *ermC* genes. IncFII plasmid pEG430-2 (GenBank accession no. LT174531.1) was 68,999 bp, harbored *ermB* and *ermC* genes downstream of an IS*6* transposase, and had a 33,429-bp DNA transfer region comprised of 37 contiguous genes (Fig. 4A). Plasmid pEG430-2 shared significant DNA homology to two other previously sequenced IncFII plasmids, p183660 (GenBank accession no. KX008967; coverage, 86%; identity, 98%) and pKSR100 (GenBank accession no. LN624486; coverage, 89%; identity, 98%), which were identified, respectively, in S. sonnei and S. flexneri serotype 3a isolates associated with disease in MSM.

Despite the erratic distribution of the *mphA* gene in the S. sonnei isolates recovered in 2000 and 2010, sequence analysis demonstrated that these isolates likely carried *mphA* on a similar IncI plasmid backbone of a comparable size. *De novo* assembly of S. sonnei DE105 effectively produced an entire plasmid sequence of 113,548 bp, and the plasmid was designated pDE105 (GenBank accession no. MG569891) (Fig. 4B). Plasmid pDE105 was analogous in size and structure to a previously described IncI plasmid, pHV292, from an Escherichia coli isolate identified in the poultry production system in Switzerland (GenBank accession no. KM377239.1). The *mphA* gene was located downstream of an IS*3*/IS*911* transposase (*orfA-orfB*) and several additional AMR genes associated with a *tnpA* transposon and conferring resistance to sulfonamides (*folP*), streptomycin (*strepAB*), β-lactams (*bla*_TEM-1_), and tetracycline (*tetA-tetR*). Plasmid pDE105 also contained a type IV secretion system with *traI* and *traJ* genes, responsible for conjugal transfer, and an operon for pilus biosynthesis (*pilI*, *pilQ*, *pilM*, *pilN*, *pilO*, and *pilP*).

We lastly performed plasmid isolation and sequencing of an additional S. sonnei isolate (DE891) which was distantly related to DE105. *De novo* plasmid assembly produced seven contiguous sequences of 115 kb that spanned 99.6% of pDE105 and that had 99% DNA sequence identity. These data confirmed a common IncI plasmid backbone within the *mphA*-positive Vietnamese S. sonnei population. Mapping the remaining *mphA* plasmid sequences against pDE105, we found that they all shared a common genetic synteny (∼90 kb) which contained the same resistance gene cassettes.

## DISCUSSION

Azithromycin is commonly thought to be a last-resort drug for the treatment of dysentery, but the increasing number of reports of decreased susceptibility to azithromycin in Shigella isolates is concerning. This problem has been observed in disparate populations, including among MSM in affluent areas and children with dysentery in LMICs. Antimicrobial options for treating infections caused by MDR and/or ciprofloxacin-resistant Shigella spp. are limited, especially for children or when an oral antimicrobial is required. In this large set of clinical Shigella species isolates collected over 18 years in Vietnam and Laos, in both of which Shigella-associated dysentery is endemic, we found a low proportion (∼5%) of Shigella isolates with decreased susceptibility to azithromycin. This low rate of nonsusceptibility may be associated with the initial low rates of nalidixic acid and ciprofloxacin resistance and, thus, limited azithromycin usage. To our knowledge, this is the largest collection of Shigella spp. exhibiting decreased susceptibility to azithromycin reported from this region. The plasmid-mediated acquisition of *mphA* and *ermB* was identified to be the principal mechanism for azithromycin resistance.

As human-restricted pathogens, Shigella spp. likely acquire resistance from the colonizing microbiota by plasmid transfer. This phenomenon has previously been demonstrated with E. coli donating *mphA* to S. sonnei (24). All of the identified *mphA*-associated plasmids have previously been described in E. coli, supporting their role as a reservoir from which AMR Shigella spp. may emerge. We demonstrate that the mechanism of azithromycin resistance in Shigella spp. arose sporadically during this period through at least seven plasmid acquisition events at different time points (from 2000 to 2009). Shigella spp. harboring azithromycin resistance plasmids appear not to have been maintained within the population, which may be associated with a lack of antimicrobial selection pressure, heterogeneity in the populations sampled, or simply the instability of the described resistance plasmids. There was only one example in the S. sonnei population in which an *mphA*-harboring plasmid subclade was maintained for at least 2 years (2000 and 2001).

Given the limited antimicrobial treatment options available for Shigella-associated dysentery and the now widespread use of azithromycin, it is critical that laboratories identify clinical isolates nonsusceptible to azithromycin. We assessed the suitability of recently published ECVs for use as clinical susceptibility breakpoints. The MIC and disc zone sizes for S. flexneri in this study were consistent with the ECV guidance proposed by CLSI for MIC and disc diffusion measurements to identify non-wild-type S. flexneri isolates, based on the detection of a resistance mechanism (16). In contrast, the distribution of MICs for azithromycin in S. sonnei was not concordant with the CLSI ECV guidance, with a skew to the right. Our data support a higher ECV and susceptibility breakpoint for S. sonnei of ≥32 mg/liter and suggest that a tentative zone size of ≤11 mm around a 15-μg azithromycin disc can identify non-wild-type isolates. These thresholds are supported by confirmatory PCR amplifications and genome sequencing, which corroborated the presence of an azithromycin resistance gene in these 22 non-wild-type isolates and demonstrated an acceptably small proportion of discrepancies according to CLSI criteria (21).

Limitations to our interpretations include the retrospective nature of the analysis of the data from the associated collection of organisms and a lack of clinical outcome data. The clinical impact of reductions in azithromycin susceptibility is uncertain, as azithromycin achieves a high concentration in intracellular compartments, such as within macrophages and colonic epithelial cells. The pathogenesis of Shigella spp. requires colonic epithelial cells for invasion, intracellular survival, and replication (8). Consequently a positive clinical outcome may be achieved even in the context of reduced *in vitro* susceptibility. Additionally, broth or agar dilution methods are the recognized standard method for MIC determination, and a previous study has demonstrated potential issues with measuring disc diffusion and Etests to determine azithromycin susceptibility (25). In a small study, Jain et al. demonstrated a double zone phenomenon for both methods and reported that broth dilution MICs corresponded to values intermediate to inner and outer zones (25). While zone size interpretation may be a limitation, we additionally performed genotypic screening of all isolates for associated resistance genes, with those results confirming our phenotypic testing results. Despite these limitations, the major strengths of our analyses include the large data set of clinical isolates, the wide range of azithromycin MICs, and the repeat testing of all isolates at a single center, thus limiting interlaboratory technical and interpretation errors.

While azithromycin resistance among Shigella spp. causing dysentery and diarrhea was not common in the 18-year period between 1994 and 2012 in the sampled locations, the increasing proportion of MDR, fluoroquinolone-resistant, and third-generation cephalosporin-resistant isolates will inevitably lead to the increasing use of azithromycin. During the sampling period, Shigella spp. with decreased susceptibility to azithromycin emerged on several separate occasions but failed to become established in the population. Azithromycin is being increasingly used for the treatment of suspected and confirmed Shigella infections in LMICs, despite limited evidence. In this study, we have developed tentative susceptibility breakpoints that we suggest should be evaluated in other locations. Correlation with proposed breakpoints and clinical outcomes in azithromycin-treated patients is a further priority. MIC and disc susceptibility breakpoints are urgently needed for the active global surveillance for azithromycin-resistant strains of Shigella spp. Assessment of new alternative treatments are also required to stay ahead of this potential public health problem.

## MATERIALS AND METHODS

### Ethics statement.

The bacterial isolates and data for this investigation originated from clinical studies approved by the scientific and ethical committees of The Hospital for Tropical Diseases (HTD) in Ho Chi Minh City, Vietnam, all other participating hospitals, and the Oxford Tropical Research Ethics Committee (OXTREC) in the United Kingdom. The study also included the characterization of bacterial isolates submitted for routine diagnostic purposes. Study participants or parents of young participants were required to provide written informed consent for the collection of samples and subsequent analyses, except when samples were collected as part of routine care.

### Study sites.

The majority of fecal specimens from which Shigella spp. were isolated were collected in a series of pediatric studies performed in Vietnam between 1994 and 2012, as previously described (6). Briefly, children presenting with either diarrhea or dysentery were recruited into observational studies (6, 20, 26) or treatment trials (27, 28) performed at The Hospital for Tropical Diseases (HTD), Children's Hospital 1, or Children's Hospital 2 in Ho Chi Minh City, Vietnam. Additional microbiology isolates collected for routine diagnostic purposes from Hué Central Hospital in Hué, Vietnam, Khanh Hoa General Hospital in Nha Trang, Vietnam, and Mahosot Hospital in Vientiane, Laos, were also included.

### Microbiology methods.

Fecal samples were collected and processed as previously described using standard microbiological methods (6, 29). Briefly, non-lactose-fermenting colonies grown on MacConkey and/or xylose lysine desoxycholate (XLD) agar (Oxoid) were identified biochemically (API 20E system; bioMérieux, Vietnam) and by slide agglutination with polyvalent somatic (O) and monovalent serotype-specific grouping antisera (from Denka Seiken, Japan, in Vietnam and Pro-Lab Diagnostics, UK, in Laos). Azithromycin susceptibility testing was performed at a single laboratory in Vietnam using the Kirby-Bauer disc diffusion method (15-μg disc) and by MIC antimicrobial gradient diffusion (Etest; AB Biodisk, Sweden), both of which were performed on Mueller-Hinton agar (Oxoid).

### Molecular methods.

Genomic DNA was extracted from S. flexneri and S. sonnei isolates using a Wizard Genomic DNA extraction kit (Promega) following the manufacturer's recommendations, with the quality and quantity being assessed using a Quant-IT kit (Invitrogen) prior to sequencing. PCR amplification for the detection of macrolide resistance genes (*mphA*, mph*B*, *ermA*, *ermB*, *ermD*, *ereA*, *ereB*, *mefA*, and *mefB*) was performed as previously described (24).

In addition, we performed a phylogenetic analysis of 247 existing S. sonnei genomes (global lineage III) and an additional 68 contemporary genomes of isolates collected during the same period (1995 to 2011) (6) (accession numbers are available in Table S1 in the supplemental material). Briefly, raw Illumina reads were mapped against an S. sonnei reference genome (the strain Ss046 chromosome [GenBank accession no. CP000038.1] and the pINV B plasmid [GenBank accession no. CP000039.1]) using the BWA program, and single nucleotide polymorphisms (SNPs) were called using SAMtools (30, 31). Phylogenetic reconstruction was performed using multiple-sequence alignment of SNPs by maximum likelihood-based phylogenetic inference (RAxML, version 8.2.8) (32) with a GTR+GAMMA substitution model. Bootstrap support for the maximum likelihood phylogeny was assessed by the use of 1,000 pseudoreplicates. The phylogenetic tree was displayed and annotated using the iTOL tool (33), highlighting the presence/absence of macrolide resistance genes over the study period among terminal taxa.

### Plasmid isolation and sequencing.

Bacterial conjugation was performed as described previously by combining representative isolates carrying *ermB* (EG430) and *mphA* (DE891) and E. coli J53 (sodium azide resistant) (34). E. coli transconjugants were selected on medium containing sodium azide (100 mg/liter) and azithromycin (24 mg/liter). *ermB*- and *mphA*-containing plasmids were extracted using a plasmid midikit (Qiagen) and sequenced using a MiSeq Illumina platform with 2× 250-bp pair-end reads. *De novo* assembly was performed using the SPADES (version 3.6.2) program and annotated using the Prokka (version 1.11) program (35, 36). The ABACAS program was used to map all the assembled contigs against a concatenated reference sequence containing the S. sonnei Ss046 chromosome (GenBank accession no. CP000038.1), virulence plasmid pSs046 (GenBank accession no. CP000039.1), and three small plasmids commonly found in S. sonnei isolates belonging to global lineage III: spA (GenBank accession no. CP000641.1), spB (GenBank accession no. CP000642.1), and spC (GenBank accession no. CP000643.1) (37). The unmapped assembled sequences were presumed to contain plasmids expressing *ermB* and *mphA*, and incompatibility (Inc) groups were then determined using *in silico* PCR by mapping the primers described previously to these unmapped sequences using an in-house script at the Sanger Institute (38). The presence of the plasmid expressing *ermB* and *mphA* was confirmed by comparing the plasmid sequences with the sequences of previously sequenced plasmids in GenBank by use of the BLASTN program, and comparative analysis was performed and the results were visualized using the ACT tool (39).

### Statistical analysis.

Statistical analysis of Shigella species isolates was limited to S. flexneri and S. sonnei isolates only, as insufficient numbers of isolates of the other species were available (Table 1). For comparisons of the proportions of nonsusceptible isolates, intermediate and resistant isolates were grouped together and their proportions were compared with the proportion of susceptible isolates using Fisher's exact test. Comparison of MIC measurements from different time periods was performed by analysis of variance and a subsequent Dunn's test with the Bonferroni correction for multiple testing, with a threshold of a *P* value of <0.05 being considered significant. To determine appropriate azithromycin breakpoints, MIC histograms were constructed and disc zone diameter breakpoints were selected using the modified error rate-bounding method of Metzler and De Haan, according to CLSI recommendations (21).

### Accession number(s).

The sequence for plasmid pDE105 has been deposited in GenBank under accession no. MG569891.

## Supplementary Material

Supplemental material
