# Supplementary material for: Azithromycin Resistance in Shigella spp. in Southeast Asia
Source: Antimicrob Agents Chemother. 2018 Mar 27;62(4):e01748-17. doi: 10.1128/AAC.01748-17 (PMC5913960; doi:10.1128/AAC.01748-17)
Supplement: Supplemental material [file supp_62_4_e01748-17__index.html]

Azithromycin Resistance in Shigella spp. in Southeast Asia — Supplemental material 

# Azithromycin Resistance in Shigella spp. in Southeast Asia

## Supplemental material

- Supplemental file 1 -

  Supplemental Table S1

  XLSX, 66K
